# A No-Reference Adaptive Blockiness Measure for JPEG Compressed Images

**DOI:** 10.1371/journal.pone.0165664

**Published:** 2016-11-10

**Authors:** Chaoying Tang, Biao Wang

**Affiliations:** College of Automation Engineering, Nanjing University of Aeronautics and Astronautics, Nanjing, China; Worcester Polytechnic Institute, UNITED STATES

## Abstract

Digital images have been extensively used in education, research, and entertainment. Many of these images, taken by consumer cameras, are compressed by the JPEG algorithm for effective storage and transmission. Blocking artifact is a well-known problem caused by this algorithm. Effective measurement of blocking artifacts plays an important role in the design, optimization, and evaluation of image compression algorithms. In this paper, we propose a no-reference objective blockiness measure, which is adaptive to high frequency component in an image. Difference of entropies across blocks and variation of block boundary pixel values in edge images are adopted to calculate the blockiness level in areas with low and high frequency component, respectively. Extensive experimental results prove that the proposed measure is effective and stable across a wide variety of images. It is robust to image noise and can be used for real-world image quality monitoring and control. Index Terms—JPEG, no-reference, blockiness measure

## Introduction

Although digital images provide great convenience to our daily life, storing and transmitting these images are problematic. Consumer cameras can produce images with size more than 12 mega pixels. Some of them can even produce images with over 20 mega pixels. The standard JPEG algorithm installed in consumer cameras are commonly used to compress these images. JPEG is a discrete cosine transform (DCT) -based compression scheme. The input image is divided into 8×8 blocks, each block being transformed independently to DCT coefficients. The DCT coefficients are then quantized using a scalar quantization matrix. At low bitrates, the DCT-coded images generally suffer from visually annoying blocking artifacts as a result of coarse quantization. It can dramatically degrade the image quality.

Over the past decades, research has been carried out to alleviate blocking effects in JPEG-compressed images. Effective measurement of blocking artifacts plays an important role in the development and evaluation of these algorithms. The most widely used point-wise comparison measures, namely mean square error (MSE) and peak signal to noise ratio (PSNR), are not very well matched to perceived visual quality [1]. A lot of effort has been put in the development of effective quality measures.

In this paper, we propose a no-reference adaptive blockiness measure for JPEG compressed images. For areas with low frequency component, we use the difference of entropies across blocks as a measure. For areas with high frequency component, we use the variation of block boundary pixel values in edge images as a measure. The final index is the weighted average of the two measures. The rest of this paper is organized as follows. Section 2 gives a short review of the related work. Section 3 introduces our blockiness measure. Section 4 reports experimental results. Section 5 offers some concluding remarks.

## Literature Review

Image quality assessment techniques can be divided into two classes: subjective assessment and objective assessment. In subjective assessment, a group of testers are required to rate a batch of images compressed under different ratios. Objective methods try to estimate the compression amount using mathematical techniques. They can be further divided into two types: full-reference and no-reference methods.

Full-reference methods give an assessment through a comparison between reference and compressed images. They mainly utilize the sensitivity of human visual system and a combination of multiple features [2]. Wang and Bovik [3] proposed a universal image quality index which models image distortion as a combination of three factors: loss of correlation, luminance distortion, and contrast distortion. In [4], the measure was generalized to the Structural SIMilarity index (SSIM) to characterize the saturation effects of the visual system at low luminance and contrast regions and to assure numerical stability.

No-reference methods directly evaluate the compressed images. They are more useful because the reference image is usually unavailable. Moorthy and Bovik [5] presented two strategies—visual fixation-based weighting, and quality-based weighting to weight image quality measurements by visual importance, and demonstrated improvements on the SSIM index in both its multi-scale and single-scale versions. Wang and Bovik [6] proposed an algorithm which evaluates blockiness by computing the FFT along the rows and columns. Pan et al. [7] proposed a method based on the fact that for images with more severe blocking artifacts, more edge pixels are arrayed horizontally and vertically. Lee et al. [8] classified blocks by analyzing block boundaries and converted boundary strength into blockiness score. Li et al. [9] presented a blockiness metric which computes the regularities of pseudo structures. In [10], Li et al. proposed a quality measure for JPEG-compressed images based on Tchebichef kernels. Golestaneh and Chandler [11] presented a measure that gets the number of zero-valued discrete cosine transform (DCT) coefficients within each block. Then it uses a quality map to represent these numbers. Freitas et. al [12] proposed a machine-learning-based method that uses the histograms of local ternary pattern (LTP) as features for the training procedure. Li et al. [13] proposed a deep-neural-network-based algorithm that extracts features using shearlet transform and evolves the features using stacked auto-encoders. Then the differences of evolved features are identified by a softmax classifier. Ren et al. [14] developed a method which uses shearlet transform to localize the distributed discontinuities induced by image degradation, and exhibits the alteration of image quality by the nature scene statistics of shearlet coefficients.

A common problem with the current blockiness measures is that none of them have adaptability to image characteristics. Different areas in an image usually have different amount of high frequency component. The compression process has different influences on these areas. This property should be considered in the blockiness measure.

## Proposed Algorithm

**Fig 1:**
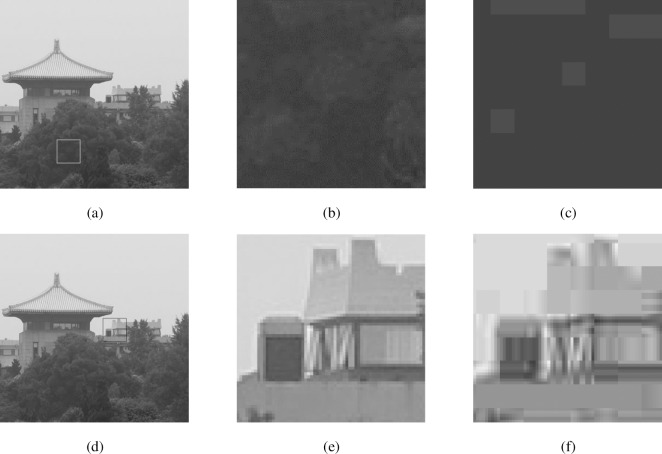
Illustration of the influence of JPEG compression on different image areas. (a) is an original image of a building, where the square shows an area with low frequency component; (d) is the same original image, where the square shows an area with high frequency component; (b) and (e) are the selected image areas in (a) and (d), respectively; (c) and (f) are the JPEG compressed versions of (b) and (e) at 0.071 bpp, respectively.

In this paper, we propose a no-reference blockiness measure which is adaptive to different areas in an image. Low frequency component in images corresponds to pixel values that change slowly over space, while high frequency component means pixel values that change rapidly. Edges in a digital image are the points at which the image brightness changes sharply or has discontinuities. Therefore sharp transients like edges represent high frequency component, while long unchanging spaces correspond to low frequency component. For the areas with low frequency component, the compression process reduces intensity variability inside a block, sometimes even generates blocks of constant gray scale, especially under high compression ratio. The block boundaries produce abrupt change of intensities across blocks. Fig 1 illustrates the influence of JPEG compression on different image areas. Fig 1(A) is an original image of a building, where the square shows an area with low frequency component (see Fig 1(B)). After compression, blocks of constant gray scale are produced (see Fig 1(C)). Comparing Fig 1(B) and 1(C), we find that before compression, the entropies of pixel values inside a block and across blocks are almost the same. However, they are quite different after compression, because of the generated block boundaries. Considering this property, we use the difference between the entropies of pixel values inside a block and across blocks to represent the compression level.

For the areas with high frequency component, the entropy difference is not influenced by the compression process, since the entropy of pixel values inside a block is already high before compression (see Fig 1D–1F). However, the generated blocks do change the edge information. Discontinuities appear around block boundaries in the edge image, and the higher the compression ratio is, the larger the discontinuities are. Therefore, in this case, we use Sobel operator to get horizontal and vertical edge images first. Then we capture the difference of pixel values around block boundaries in the edge images to represent the compression level.

Let *I* be a compressed image, and *M*_*x*_ and *M*_*y*_ be the Sobel masks defined as follows:

$$M_{x}=\left[\begin{array}{ccc} -1 & 0 & 1\\ -2 & 0 & 2\\ -1 & 0 & 1 \end{array}\right],\;M_{y}=\left[\begin{array}{ccc} -1 & -2 & -1\\ 0 & 0 & 0\\ 1 & 2 & 1 \end{array}\right].$$ (1)

The convolution results between *I* and *M*_*x*_, *M*_*y*_ are denoted as:

$$C_{x}=I*M_{x},\;C_{y}=I*M_{y},$$ (2)

where * is the convolution operator, and *C*_*x*_ and *C*_*y*_ are the horizontal and vertical edge images, respectively. Then *C*_*x*_ and *C*_*y*_ are decomposed into 8×8 non-overlapping blocks, since DCT transform is performed by 8×8 blocks in JPEG compression.

**Fig 2:**
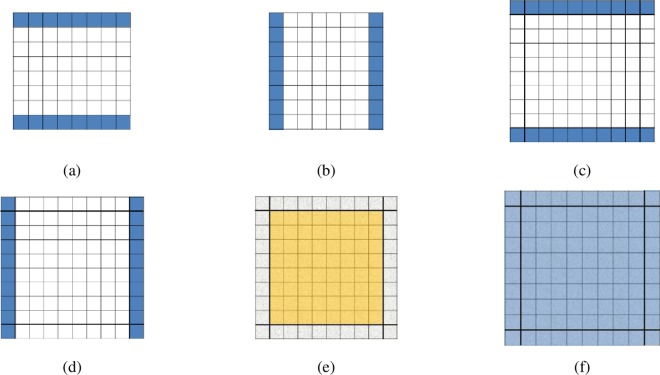
Illustration of the block areas involved in the blockiness measure. Dark elements represent the regions (a) *R*_*ih*_, (b) *R*_*iv*_, (c) *R*_*oh*_, (d) *R*_*ov*_, (e) *I*_*b*_ and (f) *I*_*bo*_.

For the *k*^th^ 8×8 block in the image, if it has edges (with high frequency component), four regions in *C*_*x*_ and *C*_*y*_, *R*_*ih*_, *R*_*iv*_, *R*_*oh*_, and *R*_*ov*_, are considered. *R*_*ih*_ and *R*_*iv*_ denote the pixels just inside the horizontal and vertical boundaries, respectively. *R*_*oh*_ and *R*_*ov*_ denote the pixels just outside the horizontal and vertical boundaries, respectively. Fig 2A–2D illustrate these regions. We define measure *s*_1_ for the areas with high frequency component. It is calculated according to the following equations:

$$s_{i}=\frac{1}{N_{i}}\left(\sum_{\left(i.j\right)\in R_{iv}}\frac{\left|C_{x}\left(i.j\right)\right|}{\max\left(C_{x}\right)}+\sum_{\left(i.j\right)\in R_{ih}}\frac{\left|C_{y}\left(i.j\right)\right|}{\max\left(C_{y}\right)}\right),$$ (3)

$$s_{o}=\frac{1}{N_{o}}\left(\sum_{\left(i,j\right)\in R_{ov}}\frac{\left|C_{x}\left(i,j\right)\right|}{\max\left(C_{x}\right)}+\sum_{\left(i,j\right)\in R_{oh}}\frac{\left|C_{y}\left(i,j\right)\right|}{\max\left(C_{y}\right)}\right),$$ (4)

$$s_{k}=\left|\frac{s_{i}^{2}-s_{o}^{2}}{s_{i}^{2}+s_{o}^{2}}\right|,$$ (5)

$$s_{1}=\frac{1}{N_{k}}\sum_{k=1}^{N_{k}}s_{k},$$ (6)

where *N*_*i*_ and *N*_*o*_ are the number of elements in the summation (here *N*_*i*_ = 32, *N*_*o*_ = 40). *N*_*k*_ is the number of blocks in this area. The measure *s*_1_ is obtained from the luminance variance of pixels inside and outside block boundaries.

For the *t*^th^ 8×8 block in the areas without edges (with low frequency component), the regions *I*_*b*_ and *I*_*bo*_ are considered. *I*_*b*_ denotes an 8×8 block, while *I*_*bo*_ represents a 10×10 pixel area with the 8×8 block in the center. Fig 2E and 2F illustrate these regions. We define measure *s*_2_ for the areas with low frequency component. It is calculated according to the following equations:

$$s_{t}=\{\begin{array}{cc} \frac{\left|En\left(I_{b}\right)-En\left(I_{bo}\right)\right|}{En\left(I_{bo}\right)}, & \text{if}\;En\left(I_{bo}\right)\neq 0\\ 0 & \text{if}\;En\left(I_{bo}\right)=0 \end{array},$$ (7)

$$s_{2}=\frac{1}{N_{t}}\sum_{t=1}^{N_{t}}s_{t},$$ (8)

where *En*(·) is the function to compute entropy. *N*_*t*_ is the number of blocks in this area. The measure *s*_2_ is obtained from the entropy difference of pixels inside and across blocks.

The final blockiness measure is defined as the weighted average of the two measures:

$$s=\frac{\sum_{i=1}^{2}w_{i}s_{i}}{\sum_{i=1}^{2}w_{i}},$$ (9)

where *w*_*i*_ (*i* = 1, 2) is the weight of the two measures which is determined as the total numbers of the two different kinds of blocks in an image.

Take Fig 1(A) as an example. After compression, *N*_*k*_, *N*_*t*_, *s*_*1*_ and *s*_*2*_ are 637, 1721, 0.220 and 0.812, respectively. In the areas with low frequency component, the average values of *En*(*I*_*b*_) and *En*(*I*_*bo*_) are 0.366 and 0.734, respectively. It can be seen that the entropy increases after compression, because of the generated block boundaries in *I*_*bo*_. The final blockiness measure *s* is 0.652.

## Experimental Results and Discussion

Extensive experiments were conducted to evaluate the performance of the proposed blockiness measure. In the first experiment, the images “sailing2” from LIVE database [15] with different levels of blocking artifacts were adopted. The bitrates of the three compressed images are 0.3929, 0.1710 and 0.1638, respectively. The subjective evaluating results of the images are represented using difference mean opinion score (DMOS). In general, the higher DMOS an image has, the lower the image quality is. Therefore a reasonable blockiness measure should be proportional to the DMOS values. The blockiness measures given by Wang’s [6], Pan’s [7], Lee’s [8], Li’s [9] and the proposed method are summarized in Table 1. It can be seen that images No. 2 and 3 have severe blocking artifacts, and image No. 3 is a little bit more serious. Therefore, the blockiness measure of No. 3 should be larger than that of No. 2. However, in Wang’s, Pan’s and Lee’s results, the score of No. 3 is smaller. It can be seen that these measures cannot accurately evaluate images with similar DMOS values. Li’s and the proposed method achieve correct results. However, the difference between Li’s scores of No. 2 and No. 3 is too large compared with the difference between the corresponding DMOS values. The scores given by the proposed method is more consistent with DMOS values. To evaluate the entropy component in the proposed measure, we calculated measure *s*_2_ in Eq (8) for the three images and put them in the last column of Table 1. It can be seen that *s*_2_ increases monotonically with the compression ratio, which means that entropy is able to describe compression level.

**Table 1:** Comparison of blockiness measures for the image“sailing2” from LIVE database with different compression levels

Image No.	Bitrates	DMOS	Wang [6]	Pan [7]	Lee [8]	Li [9]	proposed measure	*s*_2_
1	0.3929	44.81	35289.05	0.99	0.54	47.25	0.6042	0.7088
2	0.1710	59.21	103213.29	1.46	4.46	96.84	0.6945	0.9409
3	0.1638	60.08	103157.94	1.44	3.78	98.89	0.7195	0.9744

**Fig 3:**
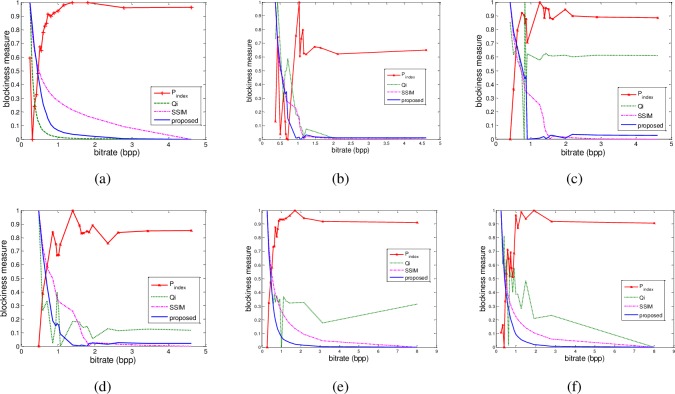
Relationship between normalized blockiness measures and bitrates of some benchmark images. (a) lena, (b) couple, (c) landscape, (d) bird, (e) lighthouse, and (f) statue. For the copyright issue, the original images are not shown here.

In the second experiment, we used six benchmark images with different themes to do evaluation. The image sizes vary from 256×256, 320×240, 512×512, to 480×720. These images were compressed under quality factors of 10, 15, 20, 25, 30, 35, 40, 45, 50, 55, 60, 65, 70, 75, 80, 85, 90, and 95, using the baseline JPEG standard in MATLAB. The corresponding bitrates can be obtained accordingly. We compared our results with a classical no-reference measure, Perra’s [2], and two full-reference measures, Qi index [3] and SSIM index [4]. Because Qi index and SSIM index give higher results for images with better quality, we use their reciprocals as blockiness indices, i.e., higher index corresponds to more serious blocking artifacts. To have a better observation, we normalize their variations to the range of 0 and 1. Fig 3 reports the experimental results. All of them have areas of different amount of high frequency component. The sensibility to the JPEG blockiness distortion was evaluated using the images compressed at different bitrates. The results show that Perra’s [2] does not change monotonically with the increase of bitrates for any of the images. In other words, its value does not represent the blockiness level in an image. Qi index has the property of monotonicity only for the image of ‘lena’ (see Fig 3(A)). For the images of ‘couple’ (Fig 3(B)), ‘bird’ (Fig 3(D)), and ‘statue’ (Fig 3(F)), the index varies drastically up and down for bitrates between 0.5 and 2.5. For the image of ‘landscape’ (Fig 3(C)) and ‘lighthouse’ (Fig 3(E)), it even increases for bitrates higher than 1. SSIM index decreases monotonically with the increase of bitrates for all the images. However, it is a full-reference measure, so it requires the original image as a reference. The proposed measure has a good property of monotonicity for all the images.

**Fig 4:**
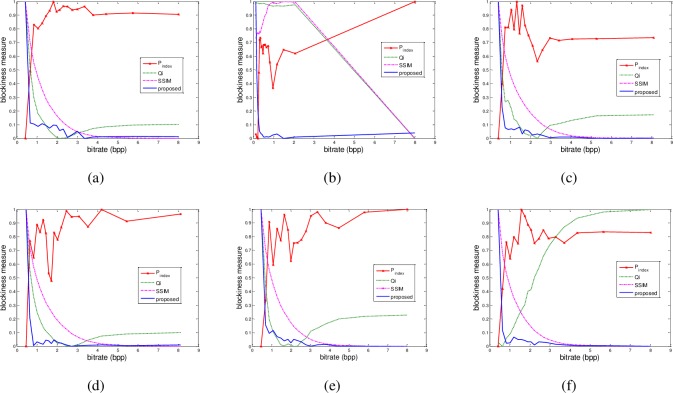
Relationship between normalized blockiness measures and bitrates of some benchmark images interfered by Gaussian noise. (a) lena, (b) couple, (c) landscape, (d) bird, (e) lighthouse, and (f) statue. For the copyright issue, the original images are not shown here.

In the third experiment, we evaluated the influence of noise on the proposed blockiness measure. The six benchmark images were adopted again. In this experiment, we added Gaussian white noise of mean 0 and variance 0.01 into the images, and then compressed them using the baseline JPEG standard in MATLAB. The following procedure was the same as in the second experiment. Fig 4 reports the experimental results. It can be seen that even SSIM does not have correct result for the image of ‘couple’ (Fig 4(B)). The proposed measure still has better performance than other no-reference measures. To evaluate whether the entropy component can correctly represent the compression level for images with different noises, we calculated measure *s*_2_ in Eq (8) for images added with Gaussian white noise of different variances and salt & pepper noise with different densities, and compressed with different levels. For the sake of space limit, we show the results of one image (“rushmore” from CSIQ database [16]) in Table 2 and Table 3. The bitrates, the blockiness measure and the corresponding *s*_2_ are shown for each variance and each compression level. It can be seen that for any case of the noises, both of the measures increase monotonically with the decrease of bitrates, which means that neither of them is interfered by noise. Therefore, the block differentiation mechanism in the proposed measure which depends on high frequency component will not be influenced by noise. In the fourth experiment, the JPEG images from four image-quality databases, including MICT[17], IVC[18], TID2008 [19], and TID2013 [20], were used to evaluate the overall performance of the proposed measure. The JPEG subsets in MICT, IVC, TID2008, and TID2013 contain 98, 60, 125, and 150 images, respectively. In these databases, 14 of 98, 10 of 60, 25 of 125, and 25 of 150 are reference images. Pearson linear correlation coefficient (PLCC) and root mean square error (RMSE) were used to evaluate the prediction accuracy, and Spearman rank-order correlation coefficient (SROCC) was used to evaluate the prediction monotonicity. These criterions were computed between the predicted scores and the subjective scores, where nonlinear fitting was first conducted to bring them on the same scale [10][21]. For comparison, the results of some popular no-reference blocking artifact metrics were also provided, including Perra’s [2], Wang’s [6], Pan’s [7], Lee’s [8], Li’s [10], Bovik’s [22], Wang’s [23], Liu’s [24], Chen’s [25], Mittal’s [26], Ye’s [27], Liu’s [28], and Liu’s [29]. A good quality measure should achieve high values in PLCC and SRCC, while low values in RMSE. Tables 4 and 5 provide the results, where the best result is marked in boldface. As shown in Table 4, the proposed measure yields the best results of both PLCC and SROCC for MICT, IVC, and TID2013. For TID2008, its SRCC is also the best, and its PLCC is very close to the best result. It can be seen that the proposed measure has a very high correlation with the subjective quality ratings. As for RMSE, the proposed measure also provides the best result for TID2008.

**Table 2:** Evaluation of the proposed measure and the corresponding *s*_2_ for the image“rushmore” from CSIQ database with Gaussian white noise of different variances and with different compression levels

Gaussian white noise of variance 0.01										
bitrates	5.5465	3.6013	3.1507	2.5128	2.0625	1.7472	1.4565	1.1735	0.8571	0.4667
proposed measure	0.1136	0.1137	0.1137	0.1137	0.1150	0.1154	0.1180	0.1185	0.1238	0.2812
*s*_2_	0.0600	0.0604	0.0609	0.0613	0.0624	0.0633	0.0648	0.0668	0.0783	0.3210
Gaussian white noise of variance 0.03										
bitrates	6.1328	4.0526	2.9955	2.7547	2.2047	1.6802	1.4709	1.2530	0.8175	0.5655
proposed measure	0.1149	0.1150	0.1156	0.1156	0.1173	0.1175	0.1186	0.1187	0.1190	0.1576
*s*_2_	0.0668	0.0670	0.0679	0.0680	0.0682	0.0689	0.0695	0.0703	0.0759	0.1386
Gaussian white noise of variance 0.05										
bitrates	6.4532	4.9935	4.2770	3.2258	2.2845	2.0960	1.6778	1.4246	1.1627	0.6384
proposed measure	0.1174	0.1180	0.1181	0.1189	0.1190	0.1192	0.1197	0.1204	0.1227	0.1316
*s*_2_	0.0697	0.0702	0.0702	0.0703	0.0706	0.0705	0.0706	0.0716	0.0722	0.0959

**Table 3:** Evaluation of the proposed measure and the corresponding *s*_2_ for the image“rushmore” from CSIQ database with salt and pepper noise of different densities and with different compression levels

salt and pepper noise of density 0.01										
bitrates	3.6381	2.5671	2.0251	1.6732	1.4392	1.2310	1.0114	0.8861	0.7511	0.4349
proposed measure	0.1195	0.1218	0.1255	0.1286	0.1305	0.1369	0.1438	0.1527	0.1625	0.2853
*s*_2_	0.0504	0.0545	0.0616	0.0689	0.0756	0.0847	0.1006	0.1148	0.1336	0.3325
salt and pepper noise of density 0.03										
bitrates	3.5220	2.7173	2.2278	1.8708	1.6075	1.3334	1.0214	0.8584	0.6801	0.4766
proposed measure	0.1213	0.1239	0.1276	0.1297	0.1349	0.1418	0.1527	0.1625	0.1906	0.2853
*s*_2_	0.0526	0.0585	0.0655	0.0712	0.0812	0.0915	0.1148	0.1336	0.1819	0.3325
salt and pepper noise of density 0.05										
bitrates	3.0583	1.8757	1.5526	1.3313	1.0866	1.0072	0.9111	0.6891	0.5635	0.4167
proposed measure	0.1248	0.1274	0.1282	0.1283	0.1291	0.1300	0.1328	0.1351	0.1496	0.2172
*s*_2_	0.0513	0.0559	0.0601	0.0627	0.0696	0.0719	0.0759	0.0903	0.1179	0.2272

**Table 4:** PLCC and SROCC of blockiness measures for the JPEG images in MICT, IVC, TID2008, and TID2013 databases.

Database	Measure	PLCC	SROCC
MICT	Perra [2]	0.7432	0.7516
	Wang [6]	0.7914	0.7689
	Pan [7]	0.8017	0.8253
	Lee [8]	0.7625	0.8097
	Li [10]	0.9107	0.9007
	Bovik [22]	0.8855	0.8067
	Wang [23]	0.9125	0.8952
	Liu [24]	0.8126	0.8136
	Chen [25]	0.8030	0.8227
	Proposed	**0.9491**	**0.9828**
IVC	Perra [2]	0.7816	0.8034
	Wang [6]	0.7112	0.7172
	Pan [7]	0.7946	0.7973
	Lee [8]	0.8982	0.8968
	Li [10]	0.9286	0.9169
	Bovik [22]	0.9049	0.9006
	Wang [23]	0.9517	0.9456
	Liu [24]	0.8295	0.8284
	Chen [25]	0.8601	0.8840
	Proposed	**0.9557**	**0.9900**
TID2008	Perra [2]	0.7263	0.0727
	Wang [6]	0.6850	0.6691
	Pan [7]	0.7062	0.5357
	Lee [8]	0.7484	0.6152
	Li [9]	0.7853	0.7401
	Li [13]	**0.9531**	0.9311
	Ren [14]	0.897	0.840
	Bovik [22]	0.7479	0.6169
	Chen [25]	0.7520	0.6866
	Proposed	0.9512	**0.9840**
TID2013	Freitas [12]	0.8877	0.7133
	Mittal [26]	0.8145	0.6567
	Ye [27]	0.8467	0.6622
	Liu [28]	0.7805	0.5922
	Liu [29]	0.8315	0.6533
	Proposed	**0.9555**	**0.9920**

**Table 5:** RMSE of blockiness measures for the JPEG images in MICT, IVC and TID2008 databases.

Database	Measure	RMSE
MICT	Perra [2]	0.8828
	Wang [6]	0.8067
	Pan [7]	0.7887
	Lee [8]	0.8538
	Li [10]	0.5452
	Bovik [22]	0.6131
	Wang [23]	**0.5399**
	Liu [24]	0.7691
	Chen [25]	0.7864
	Proposed	0.7408
IVC	Perra [2]	0.7257
	Wang [6]	0.8179
	Pan [7]	0.7063
	Lee [8]	0.5114
	Li [10]	0.4318
	Bovik [22]	0.4952
	Wang [23]	**0.3572**
	Liu [24]	0.6497
	Chen [25]	0.5935
	Proposed	0.7019
TID2008	Perra [2]	1.1708
	Wang [6]	1.2410
	Pan [7]	1.2016
	Lee [8]	1.1298
	Li [9]	0.9925
	Bovik [22]	1.1307
	Chen [25]	1.1227
	Proposed	**0.9199**

The experimental results show that the proposed blockiness measure is effective and stable across a wide variety of images. It has a good estimation of compression level over different image areas. Moreover, it is robust to image noise and can be used for real-world image quality monitoring and control.

## Conclusions

In this paper, we proposed a no-reference JPEG blockiness measure which is adaptive to the high frequency component in different image areas. It uses difference of entropies across blocks and variation of block boundary pixel values in edge images to estimate the blockiness level in an image. Extensive experiments were conducted to evaluate the performance of the proposed measure. The results indicated that compared with other blockiness indices, the proposed measure has satisfying and stable performance across a wide variety of images. It is robust to image noise and has positive perspectives in applications such as image quality monitoring and control.
